# Pilotstudie: manualmedizinische Methodenevaluation zur Modulierbarkeit des Leitsymptoms Tinnitus: Eine prospektive randomisierte Studie

**DOI:** 10.1007/s00106-022-01198-2

**Published:** 2022-08-03

**Authors:** A. Fobbe, A. Bökel, A. Lesinski-Schiedat, C. Gutenbrunner, C. Sturm

**Affiliations:** Klinik für Rehabilitationsmedizin, Medizinische Hochschule Hannover, Carl Neuberg Str. 1, 30625 Hannover, Deutschland

**Keywords:** Manuelle Therapie, Somatosensorischer Tinnitus, Halswirbelsäule, Muskeln, Ergebnisbewertung, Manual therapy, Somatosensory tinnitus, Cervical spine, Muscles, Outcome assessment

## Abstract

**Hintergrund:**

Tinnitus und Schwindel wurden schon auf vielfältige Weise untersucht. Daraus ergaben sich multiple Erklärungsansätze aus verschiedenen medizinischen Disziplinen. Auch die Muskulatur des Kiefers und der Halswirbelsäule wurde diesbezüglich erforscht. Es zeigten sich deutliche Hinweise dafür, dass bei Funktionsstörungen dieser Muskulatur Tinnitus ausgelöst werden kann. Diese Unterart des Tinnitus wird als sog. zervikogener somatosensorischer Tinnitus bezeichnet.

**Ziel der Studie:**

Das Ziel war die Untersuchung des Effekts der manuellen Therapie auf die von Probanden berichtete, individuell empfundene Beeinträchtigung durch zervikogenen somatosensorischen Tinnitus (Tinnitus Handicap Inventory), auf die Schwindelsymptomatik (Dizziness Handicap Inventory) und auf die hypertone zervikokraniale Muskulatur.

**Methodik:**

In einer prospektiven randomisierten Studie wurden 80 Patient*innen (40 in der Interventionsgruppe und 40 in der Kontrollgruppe) ärztlich untersucht und befragt. Anschließend erhielten sie manuelle Therapie.

**Ergebnisse:**

Nach manueller Therapie zeigten sich bzgl. des Tinnitus Handicap Inventory, des Dizziness Handicap Inventory und muskulärer Hypertonien signifikante Unterschiede zwischen den Gruppen zugunsten der Interventionsgruppe.

**Schlussfolgerung:**

Die manuelle Untersuchung und Therapie stellten sich als wirksam heraus. Sie sollte bei ausbleibender HNO-ärztlicher Organpathologie und Verdacht auf zervikogenen somatosensorischen Tinnitus verstärkt zur Anwendung kommen. Die Rolle der einzelnen Muskeln gilt es tiefergehend zu untersuchen.

## Abstract (en)

**Background:**

Tinnitus and vertigo have been studied in many ways. This led to a variety of explanations from multiple medical disciplines. The musculature of the jaw and cervical spine have also been researched in this regard. Dysfunctional musculature can trigger tinnitus and dizziness. This subtype of tinnitus is called cervicogenic somatosensory tinnitus.

**Objective:**

The aim of the present study was to investigate the effect of manual therapy on subject-reported, individually perceived impairment due to cervicogenic somatosensory tinnitus (Tinnitus Handicap Inventory), dizziness (Dizziness Handicap Inventory), and hypertonia of the musculature of the head and cervical spine.

**Materials and methods:**

In a prospective randomized trial, 80 patients (40 in the intervention group/40 in the control group) were medically examined and interviewed. Afterwards, they received targeted manual therapy.

**Results:**

After manual therapy, there were significant differences in the Tinnitus Handicap Inventory, Dizziness Handicap Inventory, and muscular hypertonia between the groups, all in favor of the intervention group.

**Conclusion:**

Manual examination and therapy proved to be effective. It should be increasingly applied in the absence of ENT pathology and suspected cervicogenic somatosensory tinnitus. The role of individual muscles requires further investigation.

## Hintergrund und Fragestellung

In alten Zeiten glaubten die Menschen, im Tinnitus die Götter sprechen zu hören [28]. Inzwischen gilt Tinnitus als Volkskrankheit und weniger als eigenständige Krankheit denn als Symptom. In Deutschland sind etwa 3 Mio. Menschen dauerhaft und behandlungsbedürftig betroffen. Pro Jahr kommen ca. 340.000 Neuerkrankungen hinzu [23].

Tinnitus kann akut (< 3 Monate) oder chronisch (> 3 Monate) und objektiv oder subjektiv sein. Der objektive Tinnitus resultiert aus einer körpereigenen Schallquelle (z. B. Gefäßanomalien der A. carotis) und kann auskultierbar sein. Bei dem weitaus häufigeren subjektiven Tinnitus wird die Pathologie meist im Innenohr oder in einer suboptimalen auditorischen Informationsverarbeitung lokalisiert. Oft liegt primär ein Hörverlust vor. Die zentralen Mechanismen sind bis dato nicht komplett verstanden. Ist der Patient durch sein Ohrgeräusch nicht beeinträchtigt, handelt es sich um einen kompensierten Tinnitus. Dieser wird eingeteilt in Grad 1 (kein Leidensdruck) und Grad 2 (in Stille auftretend und störend bei Stress/Belastung). Liegt ein Leidensdruck vor, spricht man von einem dekompensierten Tinnitus. Dieser führt zu dauernden privaten/beruflichen Beeinträchtigungen (Grad 3) oder zur völligen Dekompensation bis hin zur Berufsunfähigkeit (Grad 4). Des Weiteren treten bei einem dekompensierten Tinnitus Komorbiditäten wie depressive Episoden oder Angststörungen auf [10].

Laut *European Multidisciplinary Guideline For Tinnitus* aktuell erwähnte Therapieoptionen sind: Cochleaimplantate, Hörgeräte, Neurostimulation, kognitive Verhaltenstherapie, Pharmakotherapie, Tinnitus-Retraining-Therapie, „sound therapy“ (z. B. Masker), diätetische und alternative Therapien wie Akupunktur. Lediglich für die kognitive Verhaltenstherapie wurde eine hochgradige Evidenz für die Wirksamkeit bei Tinnitus und somit eine starke Empfehlung ausgesprochen. Die Pharmakotherapie z. B. hat dagegen keine Wirksamkeit gegen Tinnitus, wohl aber nachweislich gegen die tinnitusassoziierten Erkrankungen wie Depression oder Stress [2].

Ein Subtyp des Tinnitus ist der sog. zervikogene somatosensorische Tinnitus („cervicogenic somatosensory tinnitus“, CST), zu dem bisher wenige Studien vorliegen, welche die Behandlungsoption der manuellen Therapie untersuchen [16, 17, 21]. Ein somatosensorischer Tinnitus ist naheliegend, wenn sich ein Tinnitus in seiner subjektiven Lautstärke und/oder Tonhöhe durch passive oder aktive Bewegungen des Kiefergelenks, der oberen Halswirbelsäule (HWS) und/oder der begleitenden muskulären Strukturen modulieren lässt [1]. Die Trigeminus- und Spinalganglien leiten beim somatosensorischen Tinnitus afferente somatosensorische Informationen aus der Peripherie an sekundäre sensorische Neuronen im Hirnstamm weiter, insbesondere an den spinalen Trigeminuskern bzw. die Kerne der dorsalen Säule. Jede dieser Strukturen sendet nachweislich erregende Projektionen zum Nucleus cochlearis. Im Tiermodell zeigte sich demnach, dass das zentrale auditorische System (Hörbahn) durch somatosensorische Afferenzen aus der HWS- oder Kieferregion aktiviert werden kann [25, 26]. Im klinischen Alltag zeigt sich dies oft nach HWS-Distorsionen oder manualtherapeutischen Anwendungen, wenn z. B. Schädelbewegungen oder manueller Druck auf Triggerpunkte zur Modulation der Tinnitussymptome führt [15]. Ein wichtiger Einflussfaktor für die CST-Entstehung ist oftmals die kraniomandibuläre Dysfunktion [19]. Reißhauer et al. veröffentlichten bereits 2006 eine Studie, in der sich zeigte, dass es bei Tinnituspatienten typische Einschränkungen der globalen HWS-Beweglichkeit gibt und dass muskuläre Funktionsstörungen hierbei eine signifikante Rolle spielen [20].

Aktuellere Studien beschreiben die positive Auswirkung von manueller Therapie auf die CST-Symptomatik und liefern Hinweise dafür, dass somatische Störungen eine zentrale Rolle in der Tinnitusentstehung spielen können [13, 19]. Auch für Schwindel scheint es solche Hinweise zu geben [5]. Im Rahmen dieser Studie wurde Schwindel als übergreifendes Symptom aufgefasst und nicht subdifferenziert, wie beispielsweise in Benommenheit oder Drehschwindel. Zunächst sollte die generelle Einflussmöglichkeit auf Verdachtsfälle durch manuelle Therapie dokumentiert werden.

Die vorgenannten Aspekte aufnehmend, wurde die vorliegende Studie konzipiert, um bei entsprechendem Verdacht die Wirksamkeit einer manuellen Therapie auf die Symptomatik des zervikogenen somatosensorischen Tinnitus und Schwindels zu untersuchen. Dabei wurden speziell die Nackenstrecker M. semispinalis capitis und M. splenius capitis in den Fokus gerückt, da sich diese beiden im klinischen Alltag unserer Tinnitus-Sprechstunde als auffällig erwiesen hatten. Über diese Muskeln lagen im Zusammenhang mit CST bisher kaum systematische Untersuchungen vor.

In der vorliegenden Studie untersuchten wir die These H0: Durch die Anwendung von manueller Therapie (MT) resultieren keine Unterschiede zwischen der Interventionsgruppe (IG) und Kontrollgruppe (KG). H1: MT führt zu einem signifikanten Unterschied zugunsten der IG. Das Ziel war (i) die Untersuchung des Effekts der MT auf die individuelle Beeinträchtigung durch die Tinnitussymptomatik (gemessen mit dem Tinnitus Handicap Inventory; THI), und die Schwindelsymptomatik (gemessen mit dem Dizziness Handicap Inventory; DHI) und (ii) Veränderungen des Tonus der zervikokranialen Muskulatur.

## Studiendesign und Untersuchungsmethoden

### Studiendesign

Zur Beantwortung der Forschungsfragen wurden für die vorliegende prospektive randomisierte klinische Studie durch manuelle Untersuchung und Fragebogen Daten erhoben (05/2020-02/2021). Das Einverständnis der Ethikkommission (Nr. 9111_BO_S_2020) und der Datenschutzbeauftragten der Medizinischen Hochschule Hannover (MHH) lagen vor.

#### Fallzahlkalkulation

Zur Planung und Berechnung der Fallzahl wurde als Referenz eine ähnlich angelegte Studie herangezogen [9]. Die Kalkulation erfolgte anhand der zu erwartenden Veränderung des THI unter der Annahme einer Teststärke von 80 % und einer Effektstärke von d = 0,7, um im Rahmen einer Varianzanalyse mit 2 Messwiederholungen und 2 Studiengruppen Veränderungen auf dem primären Zielparameter nachweisen zu können (α = 0,05). Entsprechend dieser Vorgaben wurden pro Studiengruppe 34 Patient*innen in der Analysestichprobe benötigt. Bei Annahme eines Drop-outs von 15 % im Studienverlauf sollten pro Studiengruppe 40, also insgesamt 80 Patient*innen, rekrutiert werden. Die Fallzahlkalkulation erfolgte unter Verwendung des Programms G*Power 3.

#### Ein- und Ausschlusskriterien

Die Patient*innen wurden aus dem Patientenpool der Klinik für Rehabilitationsmedizin und der HNO-Klinik der MHH via Aufruf im MHH-Intranet, durch Aushänge und durch direkte Ansprache in den Ambulanz-Sprechstunden beider Kliniken rekrutiert. Eine fachärztliche HNO-Vordiagnostik war für unsere Fragestellung nicht explizit Voraussetzung und wurde daher nicht analysiert. Es wurden speziell die Beeinträchtigungen durch die Symptome in Selbstauskunft eruiert.

Eingeschlossen wurden volljährige Patient*innen mit:

- akutem oder chronischem Tinnitus ohne Schwindel,
- akutem oder chronischem Tinnitus mit Schwindel,
- gleichzeitigen HWS-Beschwerden,
- Modulierbarkeit des Ohrgeräuschs/Schwindels durch manuelle Stimulation der Muskulatur.

Ausgeschlossen wurden Patient*innen mit:

- objektivem Tinnitus,
- Voroperationen an Hals oder Ohr,
- bekannten Gefäßanomalien im Schädel- oder Halswirbelsäulenbereich,
- schweren kognitiven Beeinträchtigungen,
- akuten Verletzungen,
- in den letzten 8 Wochen vor Studienbeginn erhaltener manueller Therapie in der Kopf/Nacken-Region.

#### Randomisierung

Es wurden 40 Karten mit A = IG und 40 Karten mit B = KG gekennzeichnet und in 80 blickdichte identische Umschläge verpackt. Bei Studieneinschluss der Patient*innen wurde durch eine Mitarbeiterin, welche nicht in die medizinische Studiendurchführung involviert war, ein Umschlag gezogen. Somit stand die Gruppenzugehörigkeit fest.

### Studiendurchführung

Zum Studienbeginn wurden alle Patient*innen ausführlich über deren Ablauf, Ziele und Untersuchungsmethoden aufgeklärt. Ein entsprechender Informationsbogen wurde ausgegeben und mündlich erläutert. Die standardisierte Untersuchung wurde von einem orthopädischen Facharzt durchgeführt. Jeder Patient und jede Patientin willigte zur Teilnahme schriftlich ein.

Während der Untersuchung erfolgte die Feststellung und Dokumentation der HWS-Beweglichkeit unter Zuhilfenahme eines CROM-3-Geräts („cervical range of motion“, s. unten). Es wurden Muskeltonus, Druckschmerz und Modulierbarkeit des Ohrgeräuschs bzw. des Schwindels geprüft. Dafür wurden die Muskeln manuell auf Triggerpunkte und Verspannungen nach manualmedizinischem Standard untersucht [27].

Im Einzelnen wurden untersucht:

- M. splenius capitis
- M. semispinalis capitis
- M. temporalis
- M. masseter
- M. pterygoideus medialis
- Mundbodenmuskulatur
- M. trapezius pars descendens
- M. levator scapulae
- M. sternocleidomastoideus

Die Studie war als Wartegruppendesign angelegt. Die Interventionsgruppe (IG) wurde am ersten Untersuchungstag (T0) wie oben beschrieben untersucht und beantwortete drei Assessments (s. unten), um die individuellen Beschwerden zu dokumentieren. Danach erfolgte zeitnah eine Serie manuelle Therapie (MT). Nach deren Beendigung schloss sich die zweite Untersuchung (T1) mit erneutem Ausfüllen der Assessments und manualmedizinischer Untersuchung an.

**Figure 1:**
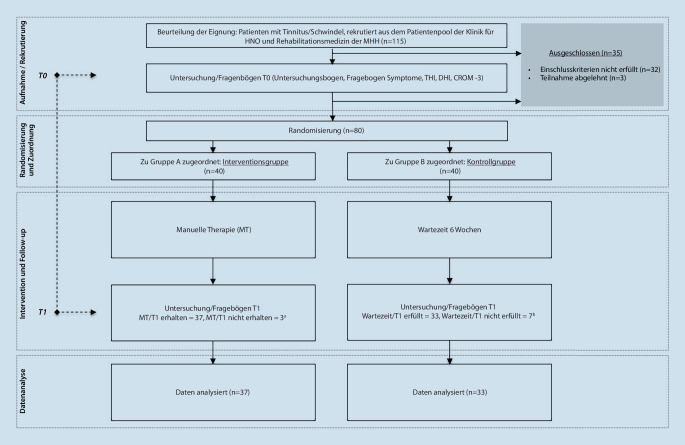

Bei der Kontrollgruppe (KG) gestaltete sich der erste Untersuchungstag (T0) genau wie bei der IG. Um zu beurteilen, ob eine spontane Befundverbesserung eingetreten war, wurde nach Ablauf von 6 Wochen eine zweite Untersuchung und Befragung durchgeführt (T1; Abb. 1). Die KG erhielt aus ethischen Gründen im Anschluss an ihre T1-Untersuchung ebenfalls eine Serie manueller Therapie wie die IG. Da der Evaluationszeitpunkt auf T1 festgelegt worden war, wurden die Ergebnisse der Therapie in der Kontrollgruppe nicht in die Auswertung eingeschlossen.

### Intervention

Die Patient*innen der Interventionsgruppe (*n* = 37) erhielten im Mittel M = 17,24 Behandlungseinheiten manuelle Therapie (min. = 13, max. = 18) in einem Zeitraum zwischen 9 und 28 Wochen. Außer der Physiotherapie-Abteilung der MHH waren noch 3 externe Fachpraxen beteiligt. Alle involvierten Physiotherapeut*innen hatten eine abgeschlossene zertifizierte Ausbildung in MT. Sie wurden vor Studienbeginn über den Ablauf und die Ziele der Studie persönlich informiert. Die angewendeten Therapien beinhalteten Übungen zur Detonisierung der hypertonen Muskeln, myofasziale Triggerpunkttherapie mit Muskel- und Bindegewebstechniken und Dehnungsübungen [27]. Des Weiteren haben die Therapeut*innen mit den Patient*innen individuelle Eigenübungen erarbeitet. Die Patient*innen wurden angewiesen, die Übungen ca. 15 min täglich durchzuführen.

#### Primärer Endpunkt.

Die Wirkung von manueller Therapie auf die Beeinträchtigung durch den Tinnitus, gemessen mit dem THI- und die Schwindelsymptomatik, gemessen mit dem DHI.

#### Sekundärer Endpunkt.

Veränderungen im Tonus der zervikokranialen Muskulatur.

Die Physiotherapeuten waren hinsichtlich der Gruppenzugehörigkeit der Patienten verblindet.

### Statistische Auswertung

Die Analyse erfolgte per Protokoll, da insbesondere Drop-outs aufgrund der Pandemiesituation zu erklären sind. Zur Untersuchung der Gruppen zum Untersuchungszeitpunkt T0 und T1 wurden Chi-Quadrat-Tests mit dem Phi-Koeffizienten für 2 Variablen verwendet (Muskeluntersuchungen).

Der Wilcoxon-Test wurde zur Berechnung der Veränderungen in den verbundenen Stichproben zu T0 und T1 angewendet. Kreuztabellen wurden genutzt, um den Korrelationskoeffizienten Cramer‑V anhand der Differenzen der Muskelwerte und der Differenzen der THI-Kategorien (T0-T1) zu berechnen. Der Mann-Whitney-U-Test wurde zur Analyse zweier unverbundener Stichproben mit ordinalen Variablen eingesetzt. Die Daten wurden mit SPSS (IBM, Armonk, NY, USA), Version 26.0 für Windows, ausgewertet.

### Material

#### Tinnitus Handicap Inventory (THI)

Der THI umfasst 25 Fragen mit 3 Unterskalen: Catastrophic Scale, Functional Scale und Emotional Scale. Die Antwortmöglichkeiten sind ja = 4 Punkte, manchmal = 2 Punkte und nein = 0 Punkte. Bei max. 100 erreichbaren Punkten erfolgt die Schweregradeinteilung in: Grad 1 = leicht 0–16, Grad 2 = mild 18–36, Grad 3 = moderat 38–56, Grad 4 = schwer 58–76 und Grad 5 = katastrophal 78–100 [11]. Die deutsche Version wurde als reliabel für klinische Studien empfohlen [7]. Eine Reduktion um 7 Punkte gilt als relevante Verbesserung in klinischen Studien, 17 Punkte Reduktion bezeichnen eine starke Verbesserung [32].

#### Dizziness Handicap Inventory (DHI)

Der DHI umfasst 25 Fragen mit 3 Unterskalen: Physical Scale, Functional Scale und Emotional Scale. Die Antwortmöglichkeiten sind ja = 4 Punkte, manchmal = 2 Punkte und nein = 0 Punkte. Bei max. 100 erreichbaren Punkten erfolgt die Schweregradeinteilung in: mild = 0–30, moderat = 31–60 und schwer = 61–100 [30]. Die deutsche Version wurde als reliabel eingestuft und für Messungen bei Schwindel empfohlen. Für den Nachweis einer Verbesserung/Verschlechterung muss sich der Score in der deutschen Version um mindestens 9, besser 16 Punkte verändert haben [8].

#### Symptomfragebogen

Dieser wurde durch die Studienleitung konzipiert. Es wurden 5 Aspekte abgefragt: bisherige Tinnitusdauer (< 3 Monate, < 6 Monate, < 12 Monate, > 2 Jahre, > 5 Jahre, > 10 Jahre) der Toncharakter (pfeifend/piepsend, brummend, klopfend, sonstige), die Lokalisation (re., li., bds.), der Schmerz im Schädel/HWS-Bereich (NRS 0–10) und die Eigeneinschätzung des Therapieerfolges nach Abschluss (0 = keine Besserung, 1 = wenig Besserung, 2 = gute Besserung, 3 = sehr gute Besserung, 4 = Symptomfreiheit).

#### Untersuchungsbogen

Dieser wurde durch die Studienleitung konzipiert. In der Untersuchung wurden dokumentiert: Inklination/Reklination, Seitneigung und Rotation der HWS in Gradzahlen (CROM-Gerät). Des Weiteren der Druckschmerz (−/+), die Hypertonie jedes der genannten Muskeln (−/+) und die Modulierbarkeit des Ohrgeräuschs und des Schwindels über den jeweiligen schmerzhaften Druckpunkt des Muskels (−/+).

#### CROM-3-Gerät

Es handelt sich um ein Kunststoffgestell zur genauen Bestimmung der HWS-Beweglichkeit, dass wie eine Brille getragen wird. Messbare Parameter sind Seitneigung, Rotation und Flexion/Extension. Die Reliabilität des CROM-3-Geräts ist in der Literatur bestätigt [31].

## Ergebnisse

Es lagen Daten von *n* = 70 Patient*innen vor im Durchschnittsalter von 48,4 Jahren (SD 13) mit 61,4 % Frauenanteil (Tab. 1). Die Gruppen zeigten zum Untersuchungszeitpunkt T0 keine signifikanten Unterschiede bezüglich des Alters, des Geschlechts sowie der Tinnitusdauer, Charakteristik oder Lokalisation, Schmerzen oder Bewegungsausmaß. Auch unterschieden sich die Gruppen nicht signifikant bezüglich des DHI (Cramer-V = 0,693; *p* = 0,342) und des THI (Cramer-V = 0,816; *p* = 0,5).

**Table 1:** 

Alter MW (Std.-Abw.)		48,4 (13)
Frauen % (*n*)		61,4 % (43)
Tinnitusdauer % (*n*)	< 3 Monate	10 (7)
	< 6 Monate	11,4 (8)
	< 12 Monate	12,9 (9)
	> 2 Jahre	20,0 (14)
	> 5 Jahre	15,7 (11)
	> 10 Jahre	30,0 (21)
Charakter % (*n*)	Pfeifend/piepsend	81,4 (57)
	Brummend	25,7 (18)
	Klopfend	1,4 (1)
	Sonstige	15,7 (11)
Lokalisation % (*n*)	Rechts	11,4 (8)
	Links	27,1 (19)
	Beidseits	61,4 (43)
THI MW (Std.-Abweichung)		43 (19)
DHI MW (Std.-Abweichung)		32,9 (16,2)

*THI* Tinnitus Handycap Inventory, *DHI* Dizziness Handycap Inventory, *MW* Mittelwert

Das von uns untersuchte Patientenkollektiv entspricht nicht dem zuvor beschriebenen Verteilungsbild der von Tinnitus betroffenen Menschen, sonst würden Altersdurchschnitt und der Anteil der Männer höher sein. Durch die offene Rekrutierung ist dies aber kein Selektionsbias. Es wurde zur Verhinderung eines solchen eine randomisierte und verdeckte Zuteilung der teilnehmenden Patienten in die Kontroll- bzw. Interventionsgruppe durchgeführt.

### Hauptzielparameter

#### THI

Die Gruppen unterschieden sich bezüglich der THI-Summenscores zu T0 nicht signifikant voneinander (U = 678,5; *p* = 0,423). Zum Zeitpunkt T1 zeigten sich allerdings signifikante Unterschiede, indem die mittleren Scorewerte in der IG signifikant niedriger lagen als bei den Kontrollen (U = 644; *p* < 0,001). Die Effektstärke liegt bei r = 0,47 und entspricht nach Cohen (1992) einem mittleren Effekt [3]. Die Mittelwerte der IG bezüglich des THI verändern sich von M_T0_ = 40,7 zu M_T1_ = 22,3 (Tab. 2). Diese Differenz von 18 Punkten im THI-Score entspricht einer starken Verbesserung nach Zeman et al. (relevante Verbesserung über 7 Punkte, starke Verbesserung über 17 Punkte Reduktion; Tab. 3; [32]).

**Table 2:** 

*Mann-Whitney-U-Test zur Untersuchung der Intergruppenunterschiede des THI-Summenscores von TO und T1 und Häufigkeiten der THI-Kategorien*				
–	T0		T1	
–	*M* = 43,0; Std.-Abw. = 19,09; *U* = 678,5; *z* = 0,801; *p* = 0,423		*M* = 31,4; Std.-Abw. = 21,72; *U* = 644; z = 3,92; *p* < 0,001; *r* = 0,47	
–	IG	KG	IG	KG
–	*n* = 37	*n* = 33	*n* = 37	*n* = 33
–	Mittlerer Rang = 33,66	Mittlerer Rang = 37,56	Mittlerer Rang = 26,49	Mittlerer Rang = 45,61
–	Mittelwert = 40,70	Mittelwert = 45,58	Mittelwert = 22,32	Mittelwert = 41,58
Grad 1: leicht % (*n*)	8,1 (3)	6,1 (2)	51,4 (19)	9,1 (3)
Grad 2: mild	35,1 (13)	36,4 (12)	27,0 (10)	36,4 (12)
Grad 3: moderat	40,5 (15)	30,3 (10)	16,2 (6)	33,3 (11)
Grad 4: schwer	16,2 (6)	18,2 (6)	5,4 (2)	9,1 (3)
Grad 5: katastrophal	0 (0)	9,1 (3)	0 (2)	12,1 (4)
*Mann-Whitney-U-Test zur Untersuchung der lntergruppenunterschiede des DHI-Summenscores von T0 und T1 und Häufigkeiten der DHI-Kategorien*				
–	T0		T1	
–	M = 32,8; Std.-Abw. = 16,26; U = 73,5; z = −0,565; *p* = 0,579		M = 21,9; Std.-Abw. = 20,70; U = 133,5; z = 2,522; *p* = 0,010; r = 0,49	
–	IG	KG	IG	KG
–	*n* = 13	*n* = 13	*n* = 13	*n* = 13
–	Mittlerer Rang = 14,35	Mittlerer Rang = 12,65	Mittlerer Rang = 9,73	Mittlerer Rang = 17,27
–	Mittelwert = 34,00	Mittelwert = 31,69	Mittelwert = 12,77	Mittelwert = 31,08
Mild % (*n*)	38,5 (5)	46,2 (6)	84,6 (11)	53,8 (7)
Moderat	61,5 (8)	46,2 (6)	15,4 (2)	38,5 (5)
Schwer	0 (0)	7,7 (1)	0 (0)	7,7 (1)

*IG* Interventionsgruppe, *KG* Kontrollgruppe

**Table 3:** 

*Häufigkeiten des THI und DHI: klinische Veränderungen von T0 zu T1 in den Gruppen und Mann-Whitney-U-Test zur Untersuchung der Gruppenunterschiede*				
	THI		DHI	
	Mann-Whitney-U = 227,000; z = −4,520; *p* < 001; *n* = 70		Mann-Whitney-U = 29,500; z = −2,826; *p* = 003; *n* = 26	
	Interventionsgruppe (*n* = 37)	Wartegruppe (*n* = 33)	Interventionsgruppe (*n* = 13)	Wartegruppe (*n* = 13)
Relevante Verbesserung % (*n*)	32,43 (12)	21,21 (7)	7,69(1)	23,08 (3)
Starke Verbesserung % (*n*)	48,65(18)	6,06 (2)	61,54 (8)	0 (0)

*THI* Tinnitus Handycap Inventory, *DHI* Dizziness Handycap Inventory

#### DHI

Die Werte des DHI waren gemäß Shapiro-Wilk-Test nicht normalverteilt (*p* > 0,5). Die Gruppen unterschieden sich bezüglich der DHI-Summenscores zu T0 nicht signifikant voneinander (U = 73,5; *p* = 0,579). Zum Zeitpunkt T1 zeigten sich demgegenüber signifikante Unterschiede zu T1 (U = 133,5; *p* = 0,010) wobei die Werte in der IG im Vergleich zur KG signifikant niedriger lagen. Die Effektstärke liegt bei r = 0,49 und entspricht nach Cohen (1992) einem mittleren Effekt ([3]; Tab. 2). Die Mittelwerte der IG bezüglich des DHI verändern sich von M_T0_ = 34,00 zu M_T1_ = 12,77. Die Differenz von 21,23 Punkten im DHI-Score entspricht einer starken Verbesserung nach Kurre et al. (relevante Verbesserung mindestens 9 Punkte, starke Verbesserung 16 Punkte Reduktion; Tab. 3; [8, 22]).

Insgesamt zeigt sich also eine signifikante Wirkung der manuellen Therapie auf die Beeinträchtigung durch den Tinnitus und die Schwindelsymptomatik mit mittlerem bis starkem Effekt.

### Nebenzielparameter

#### Muskeluntersuchungen

Die Kontrollgruppe wies zu T0 signifikant häufiger Modulierbarkeit des M. levator scapulae rechts (Phi = 0,251; *p* = 0,025) und links (Phi = 0,303; *p* = 0,007) auf, als die Interventionsgruppe. Ebenso zeigte die Kontrollgruppe signifikant mehr Häufigkeiten beim Druckschmerz des M. masseter rechts (Phi = 0,286; *p* = 0,011) im Vergleich zur Interventionsgruppe.

Zum Untersuchungszeitpunkt T1 zeigten sich deutlich häufiger signifikante Unterschiede zwischen den Gruppen bezüglich der Untersuchungsparameter Druckschmerzhaftigkeit, Hypertonus und Modulierbarkeit (Tab. 4).

**Table 4:** 

*Muskeluntersuchung: Hypertonus*								
*Chi-Quadrat-Test zur Untersuchung der Gruppenunterschiede zu T0 und T1*								
	T0				T1			
	KG	IG			KG	IG		
	Anzahl	Anzahl			Anzahl	Anzahl		
	*n* = 33	*n* = 37	Phi	*p*	*n* = 33	*n* = 37	Phi	*p*
M. splenius capitis rechts	29	34	−0,067	0,576	29	12	0,562	**<** **0,001**
M. splenius capitis links	32	36	−0,010	0,935	26	13	0,439	**<** **0,001**
M. semispinalis capitis rechts	30	28	0,202	0,091	26	8	0,571	**<** **0,001**
M. semispinalis capitis links	31	35	−0,014	0,906	26	14	0,413	**0,001**
M. temporalis rechts	6	6	0,026	0,828	3	0	0,224	0,061
M. temporalis links	6	5	0,064	0,592	1	0	0,127	0,286
M. masseter rechts	23	17	0,240	0,045	10	6	0,167	0,161
M. masseter links	21	22	0,043	0,720	16	9	0,252	**0,035**
M. pterygoideus rechts	15	13	0,105	0,379	12	3	0,334	**0,004**
M. pterygoideus links	19	17	0,116	0,331	9	4	0,211	0,077
Mundbodenmuskulatur rechts	11	14	−0,047	0,695	10	3	0,285	**0,017**
Mundbodenmuskulatur links	12	11	0,071	0,555	10	3	0,285	**0,017**
M. trapezius rechts	28	32	−0,023	0,845	29	18	0,417	**<** **0,001**
M. trapezius links	24	33	−0,211	0,077	28	12	0,529	**<** **0,001**
M. levator rechts	27	28	0,075	0,532	24	9	0,484	**<** **0,001**
M. levator links	28	30	0,050	0,676	22	9	0,426	**<** **0,001**
M. sternocleidomastoideus rechts	7	5	0,102	0,394	1	0	0,127	0,286
M. sternocleidomastoideus links	4	5	−0,021	0,862	3	0	0,224	0,061

*KG* Kontrollgruppe,* IG* Interventionsgruppe

Auch hier zeigt sich eine deutliche Wirksamkeit der manuellen Therapie im Vergleich zur Kontrollgruppe.

## Diskussion

Die Ergebnisse unserer Studie zeigen signifikante Effekte der manuellen Therapie auf die Beeinträchtigung durch den Tinnitus und die Schwindelsymptomatik bei Verdacht auf zervikogenen somatosensorischen Tinnitus.

Da eine fachärztliche HNO-Vordiagnostik keine Voraussetzung war, können wir nicht von der gesicherten Diagnose CST ausgehen, da kein Ausschluss anderer Genese stattfand. Die Modulierbarkeit der Symptome war ein wichtiges Kriterium, welches theoretisch auch bei anderen Tinnitusursachen vorliegen könnte. Eine Zuordnung zur Kategorie CST ist bei Modulierbarkeit aber laut Literatur naheliegend [1].

Nach Durchführung einer gezielten manuellen Therapie zeigen sich signifikante Verbesserungen im THI. Bezüglich der primären Fragestellung zeigte sich, dass in der IG 12 von 37 Patienten eine relevante und 18 sogar eine starke Verbesserung aufwiesen. Summiert haben also 30 der 37 Patient*innen deutlich von der Therapie profitiert. Diese Ergebnisse korrespondieren mit denen von Michiels et al. [14].

Die gleichzeitig festgestellten Veränderungen des Muskeltonus und der zervikokranialen Muskulatur sprechen für die Hypothese, dass es einen Zusammenhang zwischen den Nackenstreckern und der Tinnitussymptomatik geben könnte. Dies wird auch durch weitere Ergebnisse in der Literatur gestützt. So haben Michiels et al. festgestellt, dass bei CST-Patienten häufiger Funktionsstörungen im HWS-Bereich nachweisbar sind als bei Personen ohne CST [12]. Die Bedeutung von arthrogenen Störungen des Kiefergelenks scheint gegenüber den myogenen Dysfunktionen nachrangig zu sein. Bruxismus tritt bei Patienten mit diesen myogenen Störungen häufig mit Tinnitus vergesellschaftet auf [18].

Im Gegensatz zu älteren Voruntersuchungen, in denen besonders die Muskulatur des stomatognathen Systems genannt wurde [18], zeigte sich in unserer Untersuchung eine klare Dominanz der Nackenmuskeln mit den Nackenstreckern, dem M. trapezius und dem M. levator scapulae mit der höchsten Signifikanz und Effektstärke. So sind gerade die Ergebnisse für den M. splenius capitis und den M. semispinalis capitis bedeutsam, da es international in der Literatur zum Thema M. splenius capitis nur eine einzige uns bekannte klinische Untersuchung gab [13] und zum M. semispinalis capitis keine Untersuchung zu dieser Fragestellung. Möglicherweise ist die Bedeutung des Nackengürtels im Vergleich zu älteren Studien auch gestiegen, da diese Muskeln durch technische Entwicklungen auch stärker belastet werden. Eine Studie berechnete die hohe muskuläre Belastung bei Vorneigung des Kopfs, beispielsweise bei der Nutzung von Mobiltelefonen, und eine australische Untersuchung an Röntgenbildern zeigte schon die Entwicklung eines Knochensporns am Hinterhaupt, der vermutlich durch chronische Überlastung der Nackenstrecker entsteht [4, 24].

Wir sehen, dass die manuelle Therapie zu Verbesserungen der Muskulatur und des THI führte. Damit können wir die Null-Hypothese unserer Forschungsfrage ablehnen, nach der es keinen signifikanten Effekt der manuellen Therapie gegeben hätte. Wir leiten aus den messbaren Effekten ab, dass es einen Zusammenhang zwischen Muskulatur und Tinnitus bzw. THI geben könnte.

Die Ursachen und ihre Physiologie bezüglich des Zusammenhangs zwischen Muskeln und den Symptomen Tinnitus und Schwindel sind noch nicht hinreichend geklärt bzw. bewiesen. Eine Theorie diskutiert ein sog. Hirnstamm-Irritations-Modell, laut dem es durch vermehrte elektrische Reize aus der verspannten Muskultur zu einer Fehlverschaltung in den Hirnnervenkernen kommt und damit quasi „Kurzschlüsse“ ausgelöst werden, die zur Aktivierung im Nucleus cochlearis posterior und Nucleus vestibularis führen können [29]. Des Weiteren wurden in tierexperimentellen neuroanatomischen Untersuchungen muskuläre somatische Projektionen aus der oberen Halswirbelsäule hin zu den Cochleariskernen aufgezeigt. Dies wurde im Wesentlichen durch die Forschungsgruppe um Shore et al. erarbeitet [25, 26].

Auch bezüglich der Schwindelsymptomatik zeigte sich eine deutliche Verbesserung der angegebenen Werte im DHI. Bei 9 von 13 Patienten aus der IG, die neben dem Tinnitus auch eine Schwindelsymptomatik aufwiesen, war diese Verbesserung signifikant. Acht von ihnen wiesen dabei eine starke Verbesserung auf. Die Wichtigkeit der physiologischen Funktion der Muskulatur der oberen Halswirbelsäule für die sichere Wahrnehmung und Bewegung des Körpers im Raum ist bekannt und wurde bereits beschrieben. Ein wichtiger Aspekt dabei ist die hohe Dichte von sensorischen Elementen der oberen HWS, unter anderem in Form von Muskelspindeln [6]. Dies könnte inhaltlich zur Theorie der Reizüberflutung an den Hirnnervenkernen passen, da die vielen sensorischen Informationen bei hypertoner Nackenmuskulatur entsprechend zu einer Querverschaltung mit dem Nucleus vestibularis führen könnten. Eine eindeutige physiologische Kausalität lässt sich aber mit dem heutigen wissenschaftlichen Kenntnisstand nicht herstellen. Das klinische Bild in der manuellen Untersuchung und die Ergebnisse dieser Studie würden aber zu dieser Theoriebildung passen.

## Limitationen

Die Studie fand während der Corona-Pandemie statt. Ob es einen positiven oder negativen Einfluss gab, kann hier nicht beurteilt werden. Konkrete Einflüsse waren aber Drop-outs aufgrund von Angst vor einer SARS-CoV-2-Infektion seitens der Patienten.

Die therapeutischen Techniken waren nur in Bezug auf die erforderliche Zusatzausbildung für manuelle Therapie vorgegeben. Ob unterschiedliche Techniken zu einem unterschiedlichen Effekt auf die Muskulatur geführt haben, könnte in weiteren Untersuchungen als Folgefrage bearbeitet werden. Ebenso sollte in zukünftigen Studien die Rolle der einzelnen Muskeln näher untersucht werden. Zum Beispiel, indem man eine Patientengruppe nur im Bereich der Nackenstrecker therapiert und eine andere vergleichend nur im Bereich des M. trapezius pars descendens und M. levator scapulae oder anderen Muskeln wie zum Beispiel der Kaumuskulatur.

Eine bessere Differenzierung der Einstufung von Schmerzhaftigkeit und Hypertonie der Muskulatur wäre bei weiteren Untersuchungen wünschenswert, da hier nur dichotom (−/+) befragt wurde. Hier könnte ein System aus 0/+/++/+++ als Differenzierung helfen.

Die Beurteilung und Untersuchung erfolgten jeweils durch denselben Arzt. Dies birgt das Risiko eines Bias, erhöht aber auch die Beständigkeit in der fachlichen Einschätzung. Ein Messgerät des Drucks bis zum Auftreten eines Druckschmerzes am Muskel („tissue tension meter“) könnte hier der weiteren Objektivierung dienen.

Eine vollständige Verblindung konnte für die Probanden nicht erfolgen, da die Kontrollgruppe zum zweiten Untersuchungszeitpunkt (T1) laut Design noch keine Intervention erhalten hatte, daher also wusste, dass sie die Kontrollgruppe bildet.

Ein Bias einer „Hands-on-Therapie“, wie er im Sinne eines Placeboeffekts aus anderen Bereichen beschrieben wurde, kann auch hier nicht ausgeschlossen werden. In weiteren Untersuchungen sollte statt eines Wartegruppendesigns eine Kontrollgruppe gewählt werden, in der auch eine Form der Zuwendung wie beispielsweise edukative Gespräche oder eine Physiotherapie in vermeintlich unwirksamer Variante, wie etwa Massagetechniken im BWS- und LWS-Bereich angewendet werden.

Hörfähigkeit, bzw. Hörverluste wurden in der Analyse nicht erhoben. Da es auch einen Einfluss der Hörfähigkeit auf den Tinnitus und andersherum gibt, sollte dies in zukünftigen Studien berücksichtig werden.

## Fazit für die Praxis

Die Ergebnisse untermauern die Empfehlung, dass Untersucher bereits in der Primärdiagnostik auf zervikale muskuläre Verspannungen, speziell auch im Bereich M. splenius capitis und M. semispinalis capitis achten und entsprechende manualtherapeutische Maßnahmen einleiten.

## Abkürzungen

CST: „Cervicogenic somatosensory tinnitus“
IG: Interventionsgruppe
KG: Kontrollgruppe
Max.: Maximal
MHH: Medizinische Hochschule Hannover
Min.: Minimal
MT: Manuelle Therapie
